# Health Inequalities in Under-Five Mortality: An Assessment of Empowered Action Group (EAG) States of India

**DOI:** 10.36469/jheor.2020.18224

**Published:** 2020-12-18

**Authors:** Sarvesh Kumar, Damodar Sahu, Amit Mehto, Ravendra Kumar Sharma

**Affiliations:** 1 USMPMHS, GGS Indraprastha University, New Delhi, India; 2 National Institute of Medical Statistics, ICMR, New Delhi, India; 3 VCSG Government Institute of Medical Science and Research Srinagar, Uttarakhand

**Keywords:** concentration index, nfhs, inequality, u5mr, eag states

## Abstract

**Background:** The effect of childhood well-being programs is commonly interconnected with a change in mortality trends. The proportion of disparity shows that inequality in child mortality is more collective in the similarly evolved states than the poorer states in India.

**Objective:** To estimate and compare the health inequality of under-five mortality in Empowered Action groups (EAG) states of India.

**Methods:** Data from the National Family Health Survey (NFHS-4) was used only for the EAG States of India. Under-five mortality rates (U5MR) were calculated for associated background characteristics by using the life table method. Wealth inequality was assessed separately for all EAG states by calculating measures of concentration index (CI). Concentration curves (CC) were also plotted to see the difference in inequality.

**Results:** Higher U5MR was observed in all EAG states compared with estimates for overall India. On comparing estimates of inequality, CI values show the substantial burden of U5MR among EAG states of India. The CC shows the lowest inequality in EAG states of India.

**Conclusion:** The results suggested the need to receive various health strategy intercessions in agreement with the instance of ever-changing commitments of economic components to child health disparities in EAG states. Measuring the impact of determinants to wealth-related inequality in U5MR helps in lining up the interventions targeted at improving child survival.

## INTRODUCTION

Health inequalities are defined as differences in health status or the distribution of health determinants between different population groups. The uneven distribution may be unnecessary and avoidable as well as unjust and unfair so that the resulting health inequalities also lead to inequity in health.^1^

There have been many frameworks, policies, and programs in place both at the international level and at the national level in India to address the health inequities. The first and foremost is the sustainable development goals (SDGs), which aim to maximize population health outcomes and reduce health inequities. This forms the basis of the United Nations 2030 Agenda for Sustainable Development.^1,2^ Although all SDG goals and targets have an indirect effect on addressing the inequities, Goal 10 and Goal 5 say “Reduce inequality within and among countries” and “Achieve gender equality and empower all women and girls”.^3^

The United Nations Development Programmes Regional Human Development Report 2016 states that addressing social determinants of health and health inequities through action supporting all SDGs will improve health and well-being for all and reduce health inequities within and between countries.^4^ The social, economic and environmental conditions in which we are born, grow up, live and work are major determinants of health and well-being across the life-course.^2–4^ These conditions are often unequally distributed between individuals and societal groups, leading to unequal health outcomes. Such differences that are systematically produced by social factors (and are, therefore, preventable) are considered unfair as already stated and referred to interchangeably as social inequities in health, health inequities or health inequalities.^5^

Several focused programmes and policies have also been put in place by the Government of India to target maternal and child health directly or indirectly to reduce under-five year mortality rates (U5MR); Janani Shishu Suraksha Karyakaram (JSSK) to promote institutional deliveries by providing cashless services and entitlements;^6^ LaQshya’ programme (Labour Room Quality Improvement Initiative);^7^ Rashtriya Bal Swasthya Karyakram;^8^ Universal Immunisation;^9^ Janani Suraksha Yojana with the objective of reducing maternal and neonatal mortality by promoting institutional delivery among the poor pregnant women;^10^ Pradhan Mantri Surakshit Matritva Abhiyan to improve the quality and coverage of Antenatal Care;^11^ Navjaat Shishu Suraksha Karyakram aimed to train health personnel in basic newborn care and resuscitation;^12^ Mothers’ Absolute Affection Programme for Infant and Young Child Feeding; National Iron Plus Initiative for Anaemia Control;^13^ National Vitamin A prophylaxis Program;^14^ Integrated Child Development Services;^15^ and Mid-Day Meal Programme.^16^ The primary aim of all of these programmes is to reduce maternal and childhood mortality.

In line with all these efforts, the Ayushman Bharat or “Healthy India” is a national initiative that is part of National Health Policy 2017 to achieve the vision of Universal Health Coverage.^17,18^ However, despite all these efforts, a huge disparity exists in both the level of access to quality care^19^ and health outcomes, especially in child health, which is often measured in terms of infant mortality rate and U5MR. These differences are most prominent by geographic region, race and economic status and rural-urban difference.^20–22^ The inequalities are more pronounced in the developed states of India as compared to the less developed states. These inequalities also change with time. In some areas with favourable policies and efficient implementation, they decrease, whereas in others it increases.^21^

The inequity in health outcomes amongst children also varies. It is different in areas with different levels of development.^23^ It is evident that inequities in access and the resulting disparities in health outcome are major obstacles toward achieving SDG3, reducing U5MR, and achieving the universal health care target. In India, Empowered Action Group (EAG) states have the highest U5MR and contribute the bulk of total under-five deaths in the country.^24,25^ The U5MR in the country had declined from 109 during NFHS-1 (1992–1993), 95 in NFHS-2 (1998–1999), 74 in NFHS-3 (2005–2006) to 50 per 1000 live births in NFHS-4 (2015–2016).^26^ However, this decline throughout the years doesn’t really show a decline in disparity in child health and mortality. Thus, this study was done to unearth the level and the inequities in child health in terms of under-five mortality in the less developed EAG States of India.

### Need for the Study

An overview of demographic indicators across states within India shows that there is a wide range of discrepancies between states or even within states. State level inequalities are of particular importance in a large, populous country like India, where decision-making is decentralized as far down as the district level. Despite several national level attempts to reduce under-five mortality and to achieve SDGs, India is still far from it. The wide inequality in all these indicators makes the situation worse since inequalities in mortality rates have been documented between and within the states of India.

## MATERIAL AND METHODS

### Study Site and Population

Data from the fourth round of the National Family Health Survey (NFHS-4) were used.^27^ We considered EAG states (viz., Bihar, Chhattisgarh, Jharkhand, Madhya Pradesh, Odisha, Rajasthan, and Uttar Pradesh & Uttarakhand) including Assam. The analysis was constrained only to children born alive within five years before the interview to identify the health inequality in under-five mortality.

### Data

The NFHS-4 was conducted during 2015–2016 covering a representative sample of ever-married women aged 15–49 years to provide estimates at district, State/UTs and National level. The NFHS-4 used multi-stage sample design and collected information on fertility, family planning, infant and child mortality, maternal and child health, bio-markers, etc. (NFHS-4).^27^ In this study we considered only high focus States, 8 EAG states and Assam in view of their relatively higher mortality indicators.

### Statistical Analysis

The analysis was performed using STATA version 16.0 and Microsoft Excel. Using the birth history of the last five years, U5MR for children under various quintiles of wealth index (WI) were calculated by the Kaplan–Meier technique for survival analysis for the EAG states of India. Further, using these U5MR, concentration curve (CC) and concentration index (CI) were constructed. A p-value less than 0.05 was considered as statistically significant.

### Measures

The socioeconomic and demographic factors such as education level of the mother, caste, religion, place of residence (rural or urban), socioeconomic status, are accountable for child health inequality. For quantifying the inequality between various economic classes, a combined index named as WI was used. In NFHS-4, the WI was based on assets from a set of consumer durables, including land size, housing quality, water and sanitation facilities available to a household, and (IIPS and MACRO 2017) housing characteristics. Each household asset was assigned a weight (score) derived using principal component analysis and the sample population was separated into five subgroups or quintiles.

**Figure 1.**
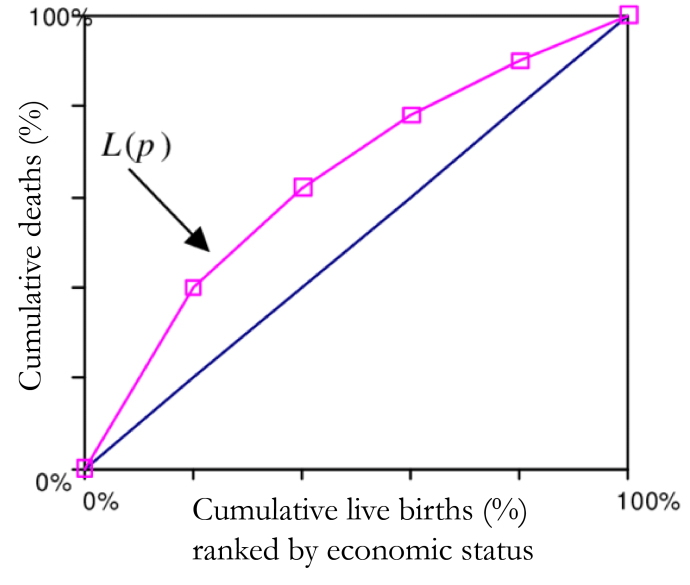
Mortality Concentration Curve

In Figure 1, the mortality CC has been demonstrated by L(p), where p shows the cumulative proportion of the study population.^28^ The y-axis depicts the cumulative proportion of under-five deaths and the x-axis shows the cumulative proportion of live births of children at risk ranked by the WI. The graph discovers the distribution of child health indicator (infant or under-five mortality) in various quintiles of socioeconomic status. The point when the curve L(p) overlaps with the diagonal implies that all children have an equivalent likelihood of death irrespective of their socioeconomic background. Whereas the area within the curve and the diagonal increases implies that inequality simultaneously increase. Along these lines, if the curve lies above the diagonal, it demonstrates inequality in under-five mortality that favours the offspring of the well-to-do class. This sort of disparity is named pro-rich. Again, if the curve lies below the diagonal then we call the inequality as pro-poor.^28–30^ However, it is not possible to find out the magnitude of inequality using this CC. The CI (Kakwani NC) inferred an index considering Gini framework to measure progressivity of social intervention.^31–33^ This index is later used to quantify the level of health inequality and named as CI. The estimation of the CI lies between −1 and +1. At the point when the CC curve is over the diagonal line then values of CI are negative and when CC is below the diagonal the values are positive. The estimation of the CI is zero when there is no inequality in wealth. When child mortality is considered as a health variable, at that point negative estimation of CI demonstrates that mortality is higher among poor children.

For the estimation of health inequality, the CI was computed utilizing an equation given by Kakwani (1997) and Wagstaff (2000).^29,32^

$$C=\frac{2}{\mu }\sum_{t=1}^{T}f_{t}\;\mu_{t}\;R_{t}-1$$

Where, C is concentration index, f_t_ μ_t_ R_t_ and μ_t_ (t = 1…, T) the mean estimation of health variable (here, mortality rate) of the t^th^ socioeconomic group. The term R_t_ is the overall position of the t^th^ socioeconomic group. In this way, the numerical expressions for μ and R_t_ are given as.

$$\mu =\sum_{t}^{T}f_{t}\;\mu_{t}$$

$$R_{t}=\sum_{\gamma =1}^{t-1}f_{\gamma }+\frac{1}{2}f_{t}$$

The term R_t_ shows the total extent of the live births up to the midpoint of each socioeconomic group.

CI does have a few limitations. To start with, CI requirements at any amount of one variable like socioeconomic status has continuous position. This forced restricted appropriateness and utilization of this index. Second, CI signifies total extents of a health variable. Thus, when mean degree of health changes then CI stays unaltered. This mirrors the viciousness of CI because of progress in mean estimation of the variable. At that point, for populations with different mean health levels we cannot think about the CI values. Lastly, CI is a proportion of relative inequality imbalance and here we cannot combine productivity with equity.^34^

## RESULTS

Table 1 showed the percentage distribution of live births according to the socio-demographic characteristics: place of residence, mother’s education, caste/tribe, religion, and wealth quintiles for EAG states of India. The majority of children were residing in rural areas, and the proportion of children in rural areas varied from about 72% in Uttarakhand to 91% in Assam. The proportion of illiterate mothers varied from about 20% in Uttarakhand to 57% in Bihar within EAG states.

**Table 1.** Percent Distribution of Live Births and Under-Five Mortality Rate (U5MR) per 1000 Live Births during the Past Five Year Period Preceding the Survey by EAG States and Assam.

**State Name**	**Percent Distribution of Live Births**																	**No. of life births**
	**Place of Residence**		**Mother's Education**				**Caste/Tribe**			**Religion**			**Wealth Index**					
	**Urban**	**Rural**	**Illiterate**	**Primary**	**Secondary**	**Higher**	**SC/ST**	**OBC**	**Others**	**Hindu**	**Muslim**	**Others**	**Poorest**	**Poor**	**Middle**	**Rich**	**Richest**	
**India**	**23.7**	**76.4**	**31.3**	**14.6**	**44.8**	**9.2**	**40.6**	**40.7**	**18.7**	**72.2**	**15.8**	**12.0**	**24.3**	**21.8**	**19.5**	**18.4**	**15.9**	**264 049**
Assam	9.1	90.9	24.4	16.3	54.9	4.4	35.5	28.8	35.7	56.1	39.1	4.8	13.3	27.7	27.5	22.9	8.7	10, 476
Bihar	10.1	89.9	56.6	12.5	27.0	4.0	26.0	59.6	14.4	83.0	16.9	0.1	39.0	26.8	17.2	11.7	5.3	25, 871
Chhattisgarh	22.9	77.1	26.6	20.3	46.0	7.1	54.0	39.4	6.6	95.4	2.3	2.3	17.4	20.8	20.9	20.4	20.5	9, 427
Jharkhand	18.6	81.4	39.4	13.3	41.3	6.1	44.2	47.6	8.3	70.1	16.0	14.0	30.2	24.6	19.6	15.7	10.0	12, 428
Madhya Pradesh	24.0	76.0	36.0	19.0	39.2	5.9	44.3	43.6	12.1	91.4	7.9	0.7	18.6	22.3	20.4	19.8	18.9	25, 088
Odisha	15.9	84.1	31.0	14.2	49.5	5.3	53.9	32.1	14.1	92.5	2.0	5.5	23.3	20.5	22.4	22.0	11.8	11, 279
Rajasthan	22.2	77.8	43.0	18.3	30.4	8.4	36.9	46.2	16.9	87.9	10.6	1.5	13.5	16.1	19.8	21.8	28.8	17, 104
Uttar Pradesh	22.0	78.0	42.8	14.3	32.7	10.1	26.4	55.5	18.1	78.1	21.7	0.3	23.0	18.8	19.2	19.1	19.8	42, 466
Uttarakhand	27.8	72.2	20.0	13.6	49.0	17.4	28.5	24.7	46.8	82.3	16.3	1.5	3.4	10.7	22.5	28.4	35.0	5, 922

Among EAG states, the proportion of mothers with higher education also varied from 4% in Bihar to about 17% in Uttarakhand. The caste wise distribution of children shows that the proportion of Scheduled Caste/Scheduled Tribe (SC/ST) varied from 26% in Bihar and Uttar Pradesh to about 54% in Odisha. The proportion of children born in Hindu families varied from the lowest 56% in Assam to the highest 95% in Chhattisgarh, whereas the children born in Muslim families varied from about 2% in Chhattisgarh to 39% in Assam.

The distribution of WI depicted that the proportion of children belonging to the poorest WI quintiles varied from about 3% in Uttarakhand to 39% in Bihar. Similarly, the proportion of children belonging to the richest quintile varied from 5% in Bihar to 35% in Uttarakhand.

Table 2 presented U5MR per 1000 live births according to socio-demographic characteristics for EAG states of India. The U5MR was higher among children residing in rural areas compared to those residing in urban areas in all EAG states; with overall India U5MR 55.3/1000 live births. The U5MR rates in rural areas varied from 54 per 1000 live births in Rajasthan to 80 per 1000 live births in Uttar Pradesh.

**Table 2.** Under-Five Mortality Rate (U5MR) per 1000 Live Births during the Past Five-Year Period Preceding the Survey by EAG States and Assam.

**State Name**	**Under-five Mortality Rate (U5MR) per 1 000 Live Births**																
	**Place of Residence**		**Mother's Education**				**Caste/Tribe**			**Religion**			**Wealth Index**				
	**Urban**	**Rural**	**Illiterate**	**Primary**	**Secondary**	**Higher**	**SC/ST**	**OBC**	**Others**	**Hindu**	**Muslim**	**Others**	**Poorest**	**Poor**	**Middle**	**Rich**	**Richest**
**India**	**39.7**	**55.3**	**67.9**	**58.9**	**42.3**	**26.6**	**56.0**	**52.8**	**41.5**	**53.2**	**53.0**	**39.8**	**71.2**	**57.2**	**49.8**	**40.9**	**28.1**
Assam	42.3	56.0	73.0	58.3	48.4	20.7	53.2	55.9	61.4	55.1	55.5	45.6	77.7	66.5	52.8	40.1	27.1
Bihar	42.0	58.9	62.0	64.4	46.1	35.6	68.8	56.0	42.9	57.9	53.4	107.1	67.5	54.2	51.8	46.0	33.9
Chhattisgarh	52.4	69.1	89.4	64.0	56.4	30.8	72.7	61.0	31.9	65.4	68.9	54.8	90.8	75.6	60.6	57.4	44.4
Jharkhand	39.2	56.4	65.1	59.6	43.4	19.0	59.0	49.2	36.9	54.9	39.7	59.4	71.9	58.0	45.1	39.5	21.4
Madhya Pradesh	57.1	64.5	70.7	71.9	54.3	28.3	73.0	56.7	45.5	63.2	58.3	28.9	84.2	64.8	71.9	54.9	36.1
Odisha	31.4	55.8	74.0	49.7	41.8	17.2	59.5	45.4	37.5	52.2	26.1	55.1	78.9	55.7	51.0	33.0	27.3
Rajasthan	35.6	54.0	57.4	57.9	37.7	35.1	57.8	49.6	33.7	49.8	51.7	40.8	64.3	61.9	53.8	48.1	34.8
Uttar Pradesh	59.5	79.9	87.6	78.0	65.1	49.4	79.9	74.7	70.8	76.3	72.8	29.3	91.4	84.7	80.4	67.1	50.0
Uttarakhand	52.5	43.1	78.4	44.5	42.2	17.4	42.8	68.5	35.9	39.4	75.0	79.4	82.1	66.8	56.8	39.7	33.1

Illiterate mothers also experienced higher under-five mortality across EAG states. Likewise, U5MR in EAG states were higher in SC/ST children. Similarly, U5MR was higher among Hindu children compared with Muslim children in most EAG states. When comparing U5MR by wealth quintile of households, it was observed first quintile (poorest) and second quintile (poorer) children have considerably higher U5MR compared with children of the richest quintile in all EAG states. The U5MR for the lowest quintile was the highest in states Uttar Pradesh (91.4), Chhattisgarh (90.8), Madhya Pradesh (84.2) and Uttarakhand (82.1), respectively. The result shows that under-five mortality sharply declined from the poorest quintile to the richest quintile in all EAG states of India.

Table 3 compared the health inequalities estimates of the CIs for U5MR separately for socio-demographic characteristics along with its standard errors (SE) CI for all EAG states of India. Under-five mortality CIs for EAG states of India were ranked according to their magnitude value of CIs. The CI values for U5MR levels constantly gave negative values, showing a heavy burden of childhood mortality among EAG states of India. It was observed that the ranking of the EAG states according to CI estimates remained almost similar by background characteristics except for wealth quintiles of households. Inequalities estimate in U5MR was most elevated in Odisha, Uttarakhand, Assam and Jharkhand and lowest in Bihar and Uttar Pradesh. We found statistically significant health inequalities estimate CIs for under-five mortality for all EAG states of India (p<0.0001).

**Table 3.** Concentration Indices and Ranks, Standard Errors for Under-Five Mortality by Background Characteristics in EAG States and Assam of India; (2015–2016)

**EAG states**	**Under-five Mortality**														
	**Place of Residence**			**Mother's Education**			**Caste/Tribe**			**Religion**			**Wealth Index**		
	**CI**	**Rank**	**SE (CI)**	**CI**	**Rank**	**SE (CI)**	**CI**	**Rank**	**SE (CI)**	**CI**	**Rank**	**SE (CI)**	**CI**	**Rank**	**SE (CI)**
**India**	**-0.162**	**-**	**0.005**	**-0.162**	**-**	**0.005**	**-0.162**	**-**	**0.005**	**-0.162**	**-**	**0.005**	**-0.162**	**-**	**0.005**
Assam	-0.168	3	0.025	-0.168	3	0.025	-0.184	2	0.027	-0.168	3	0.025	-0.152	4	0.024
Bihar	-0.076	9	0.015	-0.076	9	0.015	-0.077	9	0.015	-0.076	9	0.015	-0.082	9	0.015
Chhattisgarh	-0.129	5	0.024	-0.129	5	0.024	-0.128	4	0.024	-0.129	5	0.024	-0.126	5	0.023
Jharkhand	-0.166	4	0.023	-0.166	4	0.023	-0.165	3	0.023	-0.166	4	0.023	-0.168	2	0.023
Madhya Pradesh	-0.127	6	0.015	-0.127	6	0.015	-0.125	5	0.015	-0.127	6	0.015	-0.123	6	0.015
Odisha	-0.189	1	0.025	-0.189	1	0.025	-0.186	1	0.025	-0.189	1	0.025	-0.184	1	0.024
Rajasthan	-0.121	7	0.020	-0.121	7	0.020	-0.121	7	0.020	-0.121	7	0.020	-0.114	7	0.020
Uttar Pradesh	-0.104	8	0.010	-0.104	8	0.010	-0.104	8	0.010	-0.104	8	0.010	-0.099	8	0.010
Uttarakhand	-0.180	2	0.036	-0.180	2	0.036	-0.124	6	0.069	-0.180	2	0.036	-0.157	3	0.034

Note: p-value <0.0001 for all background characteristics. CI: Concentration Index, SE: Standard Error

**Figure 2.**
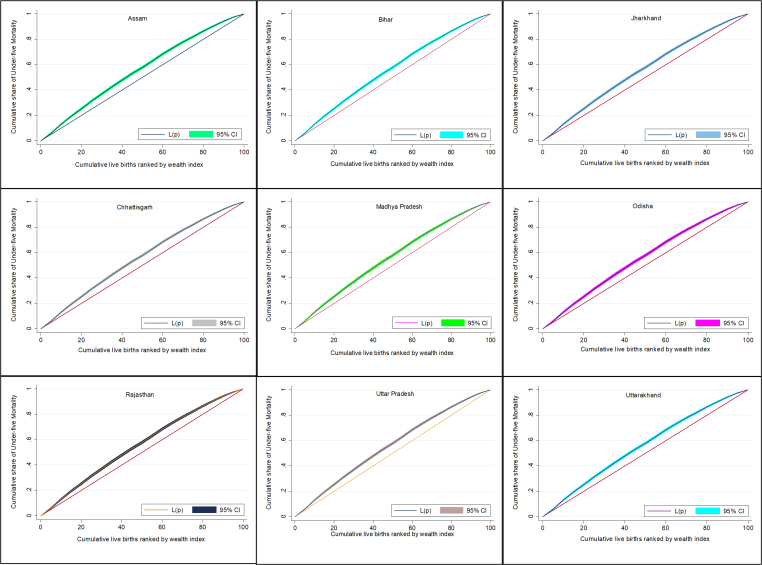
Concentration Curve of Under-Five Mortality for EAG States of India (2015–2016)

Figure 2 depicted the inequalities across the EAG states by CCs. For almost all EAG states, CCs consistently lie almost entirely above the lines of equality (45° line), which shows that under-five mortality is higher in lower wealth quintiles than in higher wealth quintiles. Thus, all CCs lie above the 45° line indicate “pro-rich” inequality (Wag staff, 2000) in mortality in all the states.^29^ The EAG states of Bihar and Uttar Pradesh show the lowest inequality compared to other EAG states’ CCs.

### Discussion

U5MR shows a lot of variation globally, nationally, and regionally, most of which could be explained by the level of development or income. This study discusses regional variation.

Using the distribution of children as per the wealth quintiles, the present study determined U5MR for important socio-demographic characteristics. The chances of survival for children born in poorer families is less, due to a myriad of reasons, including poor nutrition, poor access to health care services etc., as compared to the chances of survival of those born in better-off families. Thus, we can say that the lower to the upper quintile along with the WI shows a consistent decrease in under-five mortality. This finding is both intuitive as well as corroborated by several other studies.^35–40^ The pattern of the unequal distribution of U5MR by maternal education as reported by the present study is in line with several other studies, most notably.^41,42^

The study also showed that the most elevated inequality was seen in Odisha followed by Jharkhand and Uttarakhand. Bihar and Uttar Pradesh experienced lesser inequality in under-five mortality. Assam, Chhattisgarh, and Madhya Pradesh were in between the two extremes. These findings are corroborated in a study at the district level in Uttar Pradesh from 1991 to 2011.^43^ In this way, formative advancement in any district does not generally guarantee the improvement in child wellbeing for all socio-demographic groups of children, since inequality is the result of inequitable distribution of health resources and services and not only on the economic advancement of a state or a country, which might lead to a decrease in overall U5MR but may still widen the gap amongst the different geographical units as reported.^44,45^

All of the EAG states were found to have a negative CI indicating that there is “pro-rich” inequality in the distribution of U5MR, ranging from (-0.184) in Odisha to (-0.082) in Bihar. The same is revealed by the prominence in the CC for Odisha and a relatively flatter curve for Bihar. The remaining EAG states fall in between the two in terms of the U5MR inequality or in other words, their CIs. We also observed similar ranking across EAG states in background characteristics such as place of residence, mother’s education, and religion of under-five children.

India’s recent economic developments have not translated into improved health status of the entire populace as revealed by the differences in U5MR amongst the various socio-demographic categories in the present study. This is in part due to the above-mentioned inequitable distribution of health care services and resources and the health policies that have only recently been seen to be ‘pro-equity’ as opposed to the target-oriented policies of the past.

## CONCLUSION

Two recommendations emerge from the findings of the present study and the associated literature review. First, more research needs to be conducted to highlight the existing inequity not only in terms of health status (U5MR in this case) of the population but also in terms of health care utilization. This could be done at a state level employing more measures of inequity as done in some studies. It is only then that policymakers will be more informed and enabled to implement evidence-based policy to curb inequity of health care utilization and consequently health status, which is the second recommendation of the study, an inter-sectoral approach that factors in equity enhancing policies in all its domains.^43,46^
